# Bidirectional interactions between neuronal and hemodynamic responses to transcranial direct current stimulation (tDCS): challenges for brain-state dependent tDCS

**DOI:** 10.3389/fnsys.2015.00107

**Published:** 2015-08-10

**Authors:** Anirban Dutta

**Affiliations:** ^1^INRIA (Sophia Antipolis) – CNRS: UMR5506 – Université MontpellierMontpellier, France; ^2^Laboratoire d’Informatique de Robotique et de Microélectronique de Montpellier (LIRMM), CNRS: UMR5506 – Université MontpellierMontpellier, France

**Keywords:** transcranial direct current stimulation, electroencephalogram, near-infrared spectroscopy, hemo-neural hypothesis, neurovascular coupling

## Abstract

Transcranial direct current stimulation (tDCS) has been shown to modulate cortical neural activity. During neural activity, the electric currents from excitable membranes of brain tissue superimpose in the extracellular medium and generate a potential at scalp, which is referred as the electroencephalogram (EEG). Respective neural activity (energy demand) has been shown to be closely related, spatially and temporally, to cerebral blood flow (CBF) that supplies glucose (energy supply) via neurovascular coupling. The hemodynamic response can be captured by near-infrared spectroscopy (NIRS), which enables continuous monitoring of cerebral oxygenation and blood volume. This neurovascular coupling phenomenon led to the concept of neurovascular unit (NVU) that consists of the endothelium, glia, neurons, pericytes, and the basal lamina. Here, recent works suggest NVU as an integrated system working in concert using feedback mechanisms to enable proper brain homeostasis and function where the challenge remains in capturing these mostly nonlinear spatiotemporal interactions within NVU for brain-state dependent tDCS. In principal accordance, we propose EEG-NIRS-based whole-head monitoring of tDCS-induced neuronal and hemodynamic alterations during tDCS.

## Challenges in Clinical Translation of Transcranial Brain Stimulation—An Introduction

Transcranial direct current stimulation (tDCS)—an electrically based intervention directed at the central nervous system level—is a promising tool to alter cortical excitability and facilitate neuroplasticity (Nitsche and Paulus, 2011). However, inter-subject variability and intra-subject reliability currently limits clinical translation (Horvath et al., 2014). Indeed, a recent meta-analysis showed that the treatment effects of transcranial brain stimulation in patients with stroke are rather inconsistent across studies and the evidence for therapeutic efficacy is still uncertain (Raffin and Siebner, 2014). Here, it may be possible to reduce inter-subject variability and improve intra-subject reliability using simultaneous neuroimaging that can objectively quantify the individual brain-state before and during tDCS. Non-invasive neuroimaging techniques that have previously been combined with tDCS include electrophysiological, e.g., electroencephalogram (EEG; Schestatsky et al., 2013) and hemodynamic, e.g., functional magnetic resonance imaging (fMRI; Meinzer et al., 2014) and near-infrared spectroscopy (NIRS; McKendrick et al., 2015) approaches. Here, NIRS presents several advantages relative to fMRI, such as measurement of concentration changes in both oxygenated (HbO_2_) and deoxygenated (HHb) hemoglobin, finer temporal resolution, ease of administration and relative insensitivity to movement artifacts. Although fMRI has become the benchmark for *in vivo* imaging of the human brain, in practice, NIRS and EEG are more convenient and less expensive technology than fMRI for simultaneous neuroimaging for brain-state dependent tDCS. However, the challenge remains in modeling whole-head spatiotemporal coupling of neuronal and hemodynamic alterations induced by tDCS where such brain-state dependent tDCS need not only to consider the brain as a dynamical system but also need to consider that its parameters will be inter-individually heterogeneous, dependent on brain injury (and maladaptive plasticity, e.g., reactive gliosis, Buffo et al., 2008), task characteristics (e.g., attention issues) and other factors (Raffin and Siebner, 2014).

## Biophysical Models for Capturing Hemodynamic Alterations Induced by tDCS

Neural activity has been shown to be closely related, spatially and temporally, to cerebral blood flow (CBF) that supplies glucose via neurovascular coupling (Girouard and Iadecola, 2006). The hemodynamic response to neural activity can be captured by NIRS, which enables continuous monitoring of cerebral oxygenation and blood volume (Siesler et al., 2008). The regulation of CBF and its spatiotemporal dynamics may be probed with short-duration anodal tDCS which challenges the system with a vasoactive stimulus in order to observe the system response. Based on prior works (Nitsche and Paulus, 2000; Dutta et al., 2015), such short-duration (<1 min) anodal tDCS is postulated to cause no aftereffects and may be used to probe neurovascular coupling (and neurovascular unit, NVU; Jindal et al., 2015b). Here, CBF is increased in brain regions with enhanced neural activity via metabolic coupling mechanisms (Attwell et al., 2010) while cerebral autoregulation mechanisms ensure that the blood flow is maintained during changes of perfusion pressure (Lucas et al., 2010). During such a short-duration anodal tDCS experiment, cerebrovascular reactivity (CVR) can be measured as the change in CBF per unit change in relation to anodal tDCS intensity. Moreover, the rate of change of hemodynamic responses to same tDCS intensity may explain inter-individual differences in tDCS after-effects (Han et al., 2014). Also, phenomological model for metabolic coupling mechanisms (Attwell et al., 2010) can be used to capture CVR that represents the capacity of blood vessels to dilate during anodal tDCS due to neuronal activity-related increased demands of oxygen (Dutta et al., 2013). Here, CVR reflects the capacity of blood vessels to dilate, and is an important marker for brain vascular reserve (Markus and Cullinane, 2001). Indeed pressure–perfusion–cognition relationships may be monitored with the brain vascular reserve (Novak, 2012) where the CVR distributes CBF toward the brain areas in need of increased perfusion due to enhanced neural activity.

**Figure 1:**
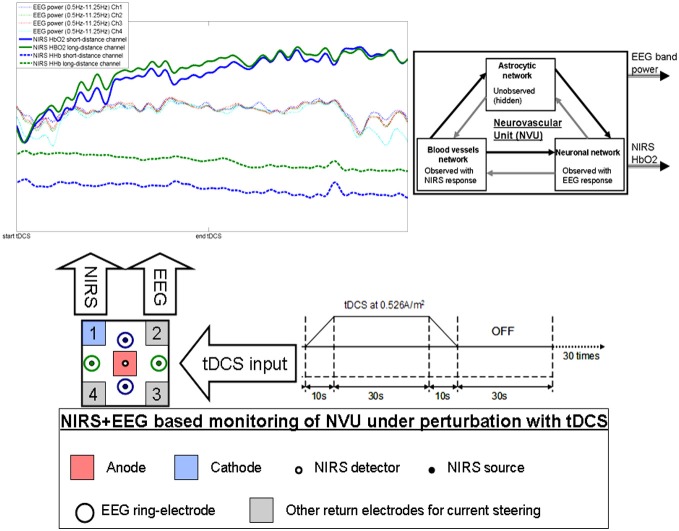
**Illustration of the effects of transcranial direct current stimulation (tDCS) from simultaneous recording of electroencephalogram (EEG) and near infra red spectroscopy (NIRS)**. The colors on the plot corresponds to the sensors. Here, only one anode and one cathode is highlighted for tDCS, however, local current steering based on NIRS-EEG feedback is possible to optimally orient the electric field with multiple return electrodes (Khadka et al., 2015). Here, neurovascular unit (NVU) consists of the endothelium, glia, neurons, pericytes, and the basal lamina in which neurons, astrocytes, and vessels are semi-independent networks operating in tandem. Neuronal network activity drives functional hyperemia via direct effects on the blood vessels network as well as indirect effects via the astrocytic network. Also, the hemodynamics changes can impact neuronal network activity via direct (diffusible messengers, electromechanical and thermal interactions) and indirect (via astrocytic network) pathways.

Prior work has shown a significant correlation between tDCS current strength and increase in regional CBF in the on-period relative to the pre-stimulation baseline (Zheng et al., 2011). We investigated regional CVR during anodal tDCS by adapting an arteriolar compliance model of the CBF response to a neural stimulus (Behzadi and Liu, 2005). Regional CVR was defined as the coupling between changes in CBF and cerebral metabolic rate of oxygen (CMRO_2_) during anodal tDCS-induced local brain activation (Leontiev and Buxton, 2007). The complex path from the tDCS-induced change of the synaptic transmembrane current, *u(t)* (only excitatory effects considered; Molaee-Ardekani et al., 2013) to a change in the concentration of multiple vasoactive agents (such as NO, potassium ions, adenosine), represented by a single vascular flow-inducing vasoactive signal, *s*, was captured by a first-order Friston’s model (Friston et al., 2000). Chander and Chakravarthy (2012) presented a computational model that studied the effect of metabolic feedback on neuronal activity to bridge the gap between measured hemodynamic response and ongoing neural activity. Here, the NVU (see Figure 1) consists of the endothelium, glia, neurons, pericytes, and the basal lamina that has been proposed to maintain the homeostasis of the brain microenvironment (Iadecola, 2004). In this connection, the role of lactate as a signaling molecule was described recently (Yang et al., 2014), which supports a (delayed) “reverse” influence in the NVU from the vessel back to neuron via lactate (Chander and Chakravarthy, 2012). Recently, a detailed biophysical model of the brain’s metabolic interactions was presented by Jolivet et al. (2015). This not only supported the astrocyte-neuron lactate shuttle (ANLS) hypothesis that the lactate produced in astrocytes (a type of glial cell) can also fuel neuronal activity but it also provided a quantitative mathematical description of the metabolic activation in neurons and glial cells, as well as of the macroscopic measurements obtained during brain imaging. Indeed, this model captured the pattern of neurovascular responses observed in rodents in response to sustained sensory stimulation where CBF only starts to increase above its baseline ~0.5–1 s after the onset of stimulation (Jolivet et al., 2015). We also found such onset effects (called “initial dip”) of anodal tDCS in stroke patients (Dutta et al., 2015). Moreover, Jolivet et al. (2015) highlighted the neuron-astrocyte cross-talk during oscillations linked to blood oxygenation levels (DiNuzzo et al., 2011) where such oscillations also occurred after anodal tDCS-based perturbation of the neuroglial networks in our EEG-NIRS stroke study (Dutta et al., 2015). We therefore postulate that short-duration anodal tDCS can be used to perturb neuroglial networks in health and disease to probe the spatiotemporal dynamics of the NVU based on simultaneous EEG-NIRS neuroimaging (Dutta, 2014; Dutta et al., 2015) and biophysical model (Jolivet et al., 2015) based analysis.

## Neural Mass or Field Models for Capturing Neuronal Alterations Induced by tDCS

During neural activity, the electric currents from excitable membranes of brain tissue superimpose at a given location in the extracellular medium and generate a potential, which is referred to as the EEG (Nunez and Srinivasan, 2006). Here, neural mass models (NMM) can provide insights into the neuromodulatory mechanisms underlying alterations of cortical activity induced via tDCS (Molaee-Ardekani et al., 2013). Specifically, the origin of tDCS-induced alterations in the EEG power spectrum was captured using a thalamocortical NMM (Dutta and Nitsche, 2013). The NMM for a single cortical source comprises of four neuronal subpopulations, excitatory pyramidal neurons (ePN), excitatory interneurons (eIN), slow inhibitory interneurons (siIN), and fast inhibitory interneurons (fiIN; Zavaglia et al., 2006). The NMM for the cortical source was coupled with another representing the thalamus (Sotero et al., 2007), which comprises of two neuronal subpopulations—an excitatory thalamocortical (eTCN) and an inhibitory reticular-thalamic (iRT). The basis of our cortical NMM is the Friston model (Moran et al., 2007) that emulates the activity of a cortical area using three neuronal subpopulations, ePN, eIN, and siIN. A population of ePN (output) cells receives inputs from inhibitory and excitatory populations of interneurons via intrinsic connections (intrinsic connections are confined to the cortical sheet). An extrinsic thalamo–cortico–thalamic loop consists of eTCN and iRT in the thalamic NMM (Ursino et al., 2010). Our lumped thalamo–cortico–thalamic network model can be used to simulate the subject-specific EEG power spectral density changes during/following tDCS (Dutta and Nitsche, 2013) by modifying the model parameters (e.g., average gain of synapses, their time constants; Zavaglia et al., 2006). We found that anodal tDCS enhances activity and excitability of the excitatory pyramidal neuron at a population level in a non-specific manner and mu-rhythm desynchronization is generated (Dutta and Nitsche, 2013). The tDCS effects on the population kinetics depend on the direction of cortical current flow determining the relative influence of acute tDCS on the cellular targets responsible for modulation of synaptic efficacy, which are primarily somata and axon terminals (Rahman et al., 2013). Basal and apical dendrites can be concomitantly polarized in opposite directions, and Layer V pyramidal neurons exhibit the highest measured somatic sensitivities to subthreshold fields (Rahman et al., 2013). Therefore, not all neural tissue will be equally affected by a given stimulation protocol which may distinctly affect neuronal populations/neuronal compartments. Indeed, a recent computational modeling study suggested that tDCS may induce opposing effects on different types of interneurons (Molaee-Ardekani et al., 2013). Here, the excitation vs. inhibition effects (Krause et al., 2013) of tDCS on the population kinetics can produce a whole spectrum of EEG signals within the oscillatory regime of a neural mass model (David and Friston, 2003).

There are several prior works that have shown both “online” effects of tDCS on EEG with EEG performed during tDCS as well as “offline” effects with EEG performed after tDCS. Here, it is important to separate studies where tDCS is applied during a rest state (Ardolino et al., 2005; Zaehle et al., 2011; Spitoni et al., 2013) or an active task state (Matsumoto et al., 2010; Mangia et al., 2014). We computationally found (Dutta and Nitsche, 2013) in concordance with the experimental results of Matsumoto et al. (2010) that tDCS effects on mu-rhythm desynchronization depend on the direction of cortical current flow determining the relative influence of acute tDCS on the cellular targets. Matsumoto et al. (2010) found that tDCS applied over the left primary motor area for 10 min at 1 mA with a 35 cm^2^ electrode influenced event-related desynchronization (ERD) during right hand grasping where the mu ERD increased after anodal tDCS and decreased after cathodal tDCS. Here, not only the “local” effects but the “distant” effects of tDCS are also relevant where Polanía et al. (2012) reported that the functional connectivity patterns significantly increased after anodal tDCS (i.e., “offline” effects) over the primary motor cortex where tDCS modulated functional connectivity of cortico-striatal and thalamo-cortical circuits. Notturno et al. (2014) showed spatial diffusion of anodal tDCS (during a motor task) effects where an increment of low alpha band power over the course of pre- and post-stimulation recording sessions was found during motor task that was localized in the sensorimotor and parieto-occipital regions. Indeed, not only the “offline” effects, but changes in functional connectivity patterns may start evolving during tDCS (i.e., “online” effects) as shown by our modeling study (Dutta and Nitsche, 2013). tDCS/EEG co-registration studies have shown that anodal tDCS mostly modulate spontaneous cortical activity in the alpha band where alpha-rhythm states have a significant effect on perceptual learning (Sigala et al., 2014). In fact, more than 60% of the observed inter-subject variability in perceptual learning can be ascribed to ongoing alpha activity where Sigala et al. (2014) highlighted the need for multidisciplinary approaches combining assessment of behavior and multi-scale neuronal activity, active modulation of ongoing brain states and computational modeling to reveal the mathematical principles of the complex neuronal interactions. We therefore postulate that concurrent EEG-NIRS-based neuroimaging of the short-duration tDCS-induced modulation can be analyzed by combining a biophysical model (Jolivet et al., 2015) of the NVU with the computational model (neural mass or field model) of multi-scale neuronal activity of the whole brain (Sigala et al., 2014) to capture the spatiotemporal dynamics of the interactions between the neuronal and hemodynamic responses in health and disease. Here, the challenges remain in ensuring the observability of the NVU with intelligent placement of EEG-NIRS sensors since presence of symmetry in the nonlinear network of NVU (see Figure 1) may decrease observability (although networks containing only rotational symmetries remain observable; Whalen et al., 2015).

## Bidirectional Interactions Between Neuronal and Hemodynamic Responses to tDCS—A Discussion

In our prior work (Dutta et al., 2015), we found an initial dip in the oxy-hemoglobin concentration and concomitant increase in the mean power spectral density within lower (<12 Hz) EEG frequency band. It was postulated that the immediate need to fuel neuronal energy recovery was via the lactate shuttle (Pellerin and Magistretti, 1994) where blood glucose supply has a longer delay (Gruetter et al., 1996). A detailed biophysical model of the brain’s metabolic interactions by Jolivet et al. (2015) also supported the ANLS hypothesis. Moreover, recent works showed that lactate can modulate the activity of primary cortical neurons through a receptor-mediated pathway (Bozzo et al., 2013) and vasomotion rhythms can influence neural firing patterns (Nikulin et al., 2014). Also, lactate promotes plasticity gene expression by potentiating NMDA signaling in neurons, and the action of lactate is mediated by the modulation of NMDA receptor activity (Yang et al., 2014). These dynamic ANLS interactions leave us to question its role in tDCS facilitated neuroplasticity and learning (Suzuki et al., 2011). Also, the spatiotemporal dynamics of the millisecond-to-second-range direct (diffusible messengers, electromechanical and thermal interactions) and seconds-to-tens-of-seconds-range indirect interaction in the NVU following tDCS, i.e., the hemo-neural hypothesis (Moore and Cao, 2008), may at least partially explain the time course of the induction of homeostatic plasticity generated by repeated tDCS of the human motor cortex (Fricke et al., 2011). Fricke et al. (2011) hypothesized a role of L-type voltage-gated Ca^2+^ channels (L-VGCC) in short-term homeostatic plasticity, since tDCS has been shown to induce a long-lasting disturbance of Ca^2+^ homeostasis (Islam et al., 1995) and induce calcium-dependent plasticity (Nitsche et al., 2003). Here, the glial network may have an important role (i.e., spatial buffering) in regulating neural activity by distributing ions (Halnes et al., 2013) in seconds-to-tens-of-seconds-range where an influence of long-lasting disturbance of Ca^2+^ homeostasis via tDCS on the myogenic and the metabolic control of cerebral circulation cannot be excluded. In fact, astrocytes, a sub-type of glia in the central nervous system, can integrate a large number of synapses and can respond to neuronal activity via neurotransmitter-evoked activation of astrocytic receptors (Araque et al., 2001). Indeed, neuronal activity can mobilize internal calcium in astrocytes and the calcium wave in different spatial–temporal dimensions can result in a higher level of brain integration (Volterra et al., 2014) where the evidence for tDCS-induced large scale changes in brain synchronization and topological functional organization has been shown after acute stimulation (Polanía et al., 2011).

**Figure 2:**
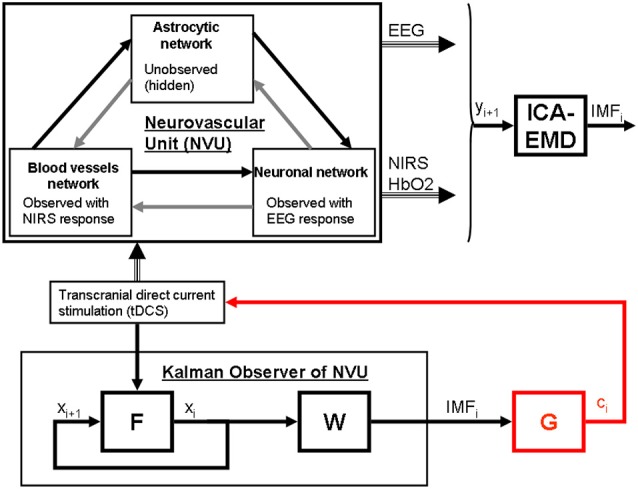
**State modulation of the NVU with (tDCS) to facilitate a brain state, e.g., spatiotemporal alpha-rhythm state**. F is the function to capture NVU system, W is the function to capture observations, ICA is independent component (IC) analysis is a linear decomposition method to transform EEG—NIRS data collected at single scalp channels to a spatially transformed “virtual channel” (i.e., a spatial filter on multi-channel EEG-NIRS data), empirical mode decomposition (EMD) is empirical model decomposition of the “virtual channel” observations that provide intrinsic mode functions (IMF) for proportional control (gain is G) of individual “virtual channel” activity or IC (e.g., posterior alpha band activity) with tDCS.

Based on these prior works, we recently proposed EEG-NIRS-based monitoring of neurovascular coupling functionality under perturbation with tDCS (Jindal et al., 2015b). Here, neuronal and hemodynamic responses measured with EEG-NIRS neuroimaging can be represented abstractly as the system response of the NVU to tDCS perturbation (see Figure 1) where presence of symmetry in the nonlinear network of NVU (see Figure 1) may decrease observability (Whalen et al., 2015). Since no real-world network has exact symmetries so with intelligent placement of EEG-NIRS sensors (e.g., to avoid systemic interference; Sood et al., 2015) along with system identification and parameter estimation techniques, it may be possible to track the spatiotemporal change of the states of the NVU. This observer model can then be used to drive multi-electrode tDCS (Dmochowski et al., 2011) for active spatiotemporal modulation of the brain states (e.g., posterior alpha-rhythm). Here, we base our discussions on the recent advances in Kalman filtering approaches to spatiotemporal nonlinear systems (Schiff and Sauer, 2008) and an understanding from group representation theory in controller or observer design by obtaining a modal decomposition into decoupled controllable and uncontrollable (observable and unobservable) subspaces (Whalen et al., 2015). Specifically, Schiff and Sauer (2008) showed the feasibility of unscented Kalman filter (UKF) for recursive estimation of system state for nonlinear systems, including unobserved variables and parameter tracking, in a spatiotemporal model of cortex where such a nonlinear system is controllable using an adaptive feedback electrical field. Here, discretization of the whole-brain detailed biophysical model of NVU (Jolivet et al., 2015), for example with Galerkin methods that are used quite robustly in fluid dynamics, will be necessary where each discrete element corresponds to a volume of tissue imaged as well as stimulated with the EEG-NIRS/tDCS unit (see Figure 1). As an alternative to a fundamental NVU model (Jolivet et al., 2015) for the volume of tissue imaged and stimulated with EEG-NIRS/tDCS unit, we tried (Dutta et al., 2015) to find an empirical model where we performed empirical mode decomposition (EMD) and the Hilbert spectrum (Huang et al., 1998) to model the system dynamics and found a negative cross-correlation between one of the intrinsic mode function (IMF) of the HbO_2_ time-series and log-transformed mean-power time-course of EEG primarily within 0.5–11.25 Hz frequency band (i.e., one of the EEG IMFs). In principal accordance, for whole-head monitoring, we propose independent component analysis (ICA) to transform multi-channel EEG-NIRS/tDCS unit imaging data to a spatially transformed “virtual channel” (i.e., a spatial filter; Jung et al., 2001). Then, the “virtual channel” activity (e.g., posterior alpha band activity) can be subjected to EMD (i.e., a temporal filter) to reduce the dimension of the observable dynamics (i.e., further observer model reduction) before developing the Kalman filter observer (Schiff and Sauer, 2008) using the IMFs. For EEG-NIRS-based monitoring of NVU under perturbation with tDCS, as shown in the Figure 2, the NVU dynamics is captured by the function F and the IMF observations by the function W. The UKF approach should match the nonlinear IMF dynamics up to the second order statistics where the feasibility remains to be tested experimentally in future studies. Furthermore, it may be possible to use the Kalman observer to calculate proportional control (see Figure 2) of the brain-state (e.g., cortical excitability; Jindal et al., 2015a) with tDCS. Here, intelligent placement of EEG-NIRS sensors and tDCS effectors is necessary to ensure observability and controllability (Whalen et al., 2015) where Whalen et al. (2015) suggested in general that more direct incoming connections into an observed node lead to higher observability and more direct outgoing connections from a controlled node lead to higher controllability. Furthermore, controllability may be enhanced with multi-modal non-invasive brain stimulation (NIBS), e.g., with direct electrical stimulation (Pulgar, 2015) and photobiostimulation (Gonzalez-Lima and Barrett, 2014), which needs to be investigated.

Towards such brain-state dependent tDCS, the challenges include the nature of observability and controllability in whole-brain complex NVU networks as well as the subtleties of the tDCS interaction with the whole-brain NVU (e.g., based on heterogeneous geometrical characteristics, Molaee-Ardekani et al., 2013) that can also have multi-timescale cross-talk and resulting complex non-linear dynamics (Jolivet et al., 2015) where the spatiotemporal observability and controllability remains to be verified in future studies.

## Conflict of Interest Statement

The author declares that the research was conducted in the absence of any commercial or financial relationships that could be construed as a potential conflict of interest.
